# Trajectories of blood pressure measures and the incidence of electrocardiographic abnormalities: a worker-based cohort study

**DOI:** 10.3389/fpubh.2026.1930829

**Published:** 2026-09-10

**Authors:** Jiahui Liang, Peitian Gao, Xuehong Liang, Jiachun Jin, Yan Jiang, Weijie Jiang, Chuifei Zhong, Yongshun Huang

**Affiliations:** 1 Guangdong Province Hospital for Occupational Disease Prevention and Treatment, Guangzhou, Guangdong, China; 2 School of Public Health, Guangzhou Medical University, Guangzhou, Guangdong, China; 3 School of Health Management, Guangzhou Medical University, Guangzhou, Guangdong, China; 4 School of Public Health, Guangdong Pharmaceutical University, Guangzhou, China; 5 Department of Labor Hygiene, School of Public Health, Shanxi Medical University, Taiyuan, China

**Keywords:** blood pressure, ECG abnormalities, occupational exposure, trajectories, workers

## Abstract

**Background:**

Evidence on the association between longitudinal blood pressure trajectories and incident electrocardiographic abnormalities among factory workers is limited, and whether occupational exposure modifies this association remains unclear.

**Methods:**

Using health examination data collected between 2016 and 2024, we applied Group-based trajectory modeling (GBTM) to identify trajectories of systolic blood pressure (SBP), diastolic blood pressure (DBP), mean arterial pressure (MAP), mid-blood pressure (Mid-BP), pulse pressure (PP), and pulse pressure index (PPI). Cox proportional hazards regression models were used to evaluate the associations between blood pressure trajectories and incident electrocardiographic (ECG) abnormalities. Stratified analyses and interaction tests were further performed to assess the modifying effects of occupational exposure.

**Results:**

Among 2,561 workers (mean age 34.65 years), 1,183 (46.2%) developed at least one ECG abnormality during the 4-year follow-up. Two trajectory patterns were identified for each blood pressure indicator. After controlling for age, gender, years of service, job type, work location, smoking status, ownership type, occupational exposure and company size, Cox regression showed HRs of 1.17 (95% CI: 1.04–1.31) for the persisting high SBP trajectory and 1.17 (95% CI: 1.04–1.32) for the increasing Mid-BP trajectory. Stratified analyses showed significant interactions of high temperature exposure with SBP and Mid-BP trajectories on ECG abnormalities (all *p* for interaction <0.05).

**Conclusion:**

The increasing Mid-BP and the persisting high SBP trajectories are prospectively associated with an increased risk of ECG abnormalities among factory workers, with high temperature exposure significantly modifying these associations. Regular monitoring of these trajectories constitutes a viable approach for workplace risk assessment and targeted cardiovascular prevention.

## Introduction

Electrocardiographic (ECG) abnormalities, including arrhythmia, represent a significant and growing component of the global disease burden. As the most common sustained arrhythmia, atrial fibrillation (AF) exemplifies this burden: the global prevalence of AF has more than doubled over the past three decades, reaching 52.6 million cases in 2021, and is projected to continue rising through 2050 (1–3). Consequently, reducing ECG abnormalities risk is an important public health priority.

Hypertension is a major contributing factor to the onset of arrhythmia (4), affecting approximately a quarter of Chinese adults (5). However, the prevalence of hypertension may be higher and its onset may occur at an earlier age, owing to complex working environments involving exposure to high temperatures, dust, and noise, which contribute to diverse and complex occupational hazard profiles (6–10).

A recent meta-analysis of 68 cohort studies reported that individuals with hypertension had a 1.50-fold higher risk of AF (95% CI: 1.42–1.58) compared with normotensive individuals (11). However, the generalizability of these findings is limited, as 44 included studies had mean participant ages above 60 years, restricting applicability to younger and middle-aged populations. Among the remaining studies, most were based on community cohorts or health screening populations, with limited representation of occupational groups. Furthermore, nearly all included studies relied on single time-point blood pressure measurements, which may oversimplify the dynamic nature of blood pressure changes over time and fail to capture the cumulative impact of sustained or progressive elevation. This highlights the need for longitudinal trajectory analyses to better understand how patterns of blood pressure change are associated with the risk of ECG abnormalities.

To date, existing studies have investigated blood pressure (BP) trajectories in relation to cardiovascular outcomes (12–14). However, evidence remains largely derived from community-based cohorts, leaving a critical gap in understanding these relationships in occupational settings. Furthermore, few studies have examined the potential modifying role of occupational exposures, limiting our understanding of how workplace environments may influence the association between BP trajectories and ECG abnormalities. Given that China remains in an era of large-scale manufacturing, understanding these associations carries substantial public health implications for developing evidence-based workplace health policies and preventive strategies. Therefore, we aimed to evaluate the association between BP trajectories and the risk of ECG abnormalities in a worker-based cohort and to investigate whether occupational exposures modify these associations.

## Methods

### Study design and participants

This study utilized data from medical institutions within Guangdong Province holding occupational health examination qualifications, collected between 2016 and 2024, among factory workers in southern China. The study consisted of two phases: (1) identifying distinct trajectories of six blood pressure measures using data from 2016, 2018 and 2020; (2) assessing the association between these trajectories and the risk of ECG abnormalities during the 2021–2024 follow-up period.

**Figure 1:**
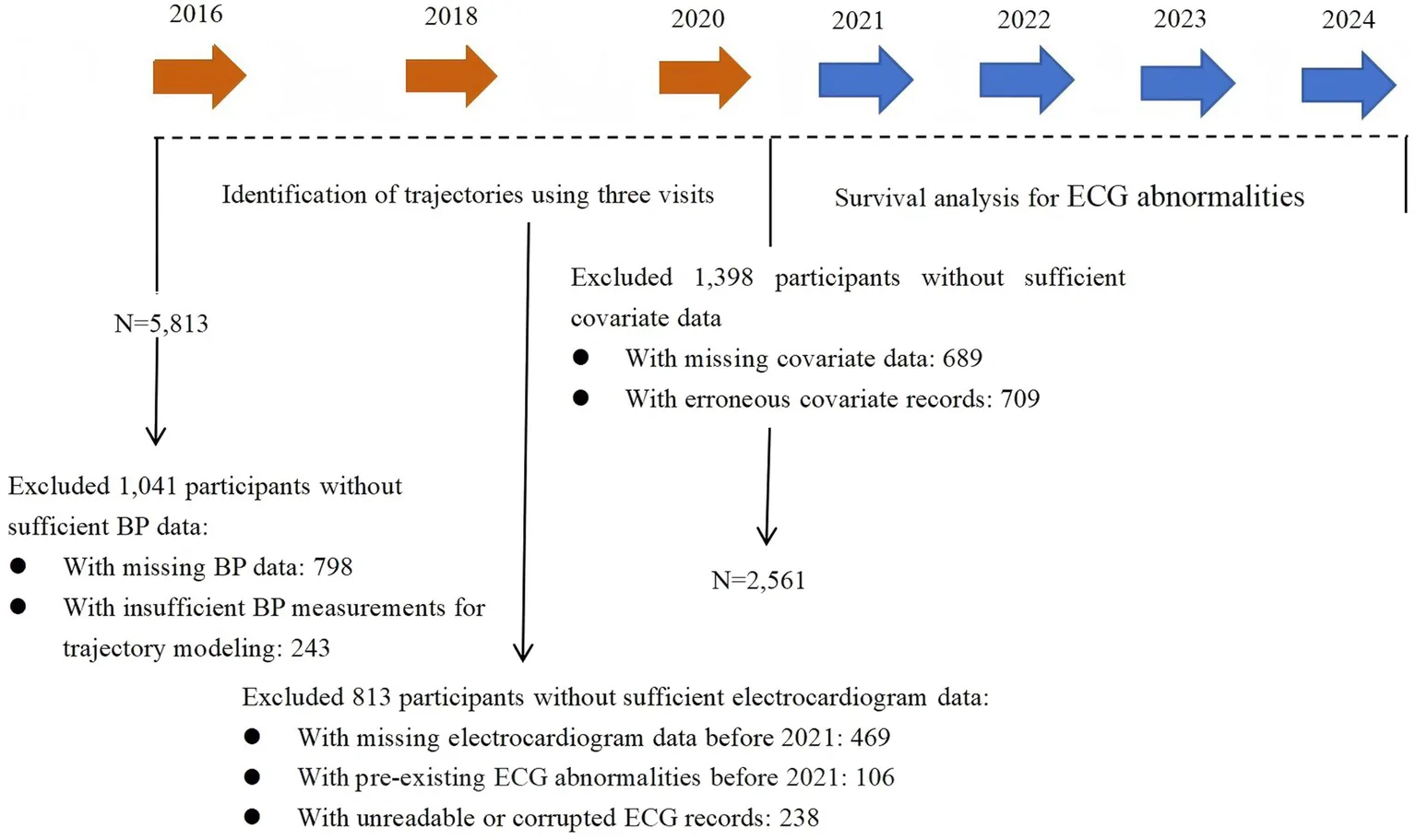
Study flowchart.

A total of 5,813 workers, with health examination records throughout the 2016–2024 period, were initially identified. We first excluded 1,041 workers lacking complete blood pressure data, and then excluded 813 with insufficient electrocardiogram data or those diagnosed with ECG abnormalities before January 1, 2021. Finally, after excluding 1,398 workers with missing covariate data or erroneous covariate records, 2,561 individuals were included in the final analysis. Compared with participants excluded from the analysis, those included had a higher proportion of public ownership and a lower proportion of dust exposure, while age, gender, and years of service were similar between the two groups (Supplementary Table S2). The complete study design and participant flow are illustrated in Figure 1.

### Assessment of blood pressure

Trained investigators assessed workers’ systolic and diastolic blood pressure using validated electronic sphygmomanometers in quiet and comfortable rooms. Workers were instructed to refrain from coffee, tea, alcohol, cigarette smoking, and vigorous exercise for a minimum of 30 min before blood pressure measurement. Blood pressure was assessed three times in a seated position on the right upper arm following a 5-min rest, with a 2-min interval between readings. The average of the last two readings was used for analysis.

To comprehensively assess blood pressure status, four derived indices were computed from systolic blood pressure (SBP) and diastolic blood pressure (DBP): mean arterial pressure (MAP) = DBP + (SBP − DBP)/3, mid-blood pressure (Mid-BP) = (SBP + DBP)/2, pulse pressure (PP) = SBP − DBP, and pulse pressure index (PPI) = PP/SBP (15, 16).

### Assessment of ECG abnormalities

The primary outcome of this study was incident electrocardiographic (ECG) abnormalities, identified through annual occupational health examinations between 2021 and 2024.

All ECG examinations were performed by trained technicians using standard 12-lead electrocardiographs. After a 5-min rest in a supine position, standard 12-lead ECGs were recorded. The ECG machine automatically generated a preliminary diagnostic report. All ECG tracings and preliminary reports were reviewed and confirmed by at least one qualified occupational health physician. Disagreements were resolved through consensus or consultation with a senior cardiologist. The final diagnosis was recorded in the occupational health examination system. The interpreting physicians were blinded to participants’ blood pressure trajectories.

Incident ECG abnormalities were defined as any abnormal ECG findings during follow-up, including but not limited to arrhythmias (e.g., atrial fibrillation, bradycardia, tachycardia, conduction block, premature contractions, and pre-excitation syndrome) and non-specific ECG changes (e.g., ST-T changes, axis deviation, left ventricular high voltage, and low QRS voltages in limb leads).

### Covariates

Covariates were obtained from self-reported questionnaires and factory records at baseline (2021). Self-reported information included age, gender, years of service, job type, work location, and smoking status. Factory records provided data on ownership type, occupational exposure and company size. Age was categorized into four groups: 20–29, 30–39, 40–49, and 50–59 years. Gender was classified as male or female. Job type was classified as management, social service worker, manufacturing worker, professional technical worker, or other. Work location was categorized as urban or rural. Ownership type was grouped as public, non-public, mixed, or other. Company size was categorized as small, medium, or large. Smoking status was defined as never, former, or current. Occupational exposures, including high temperature, dust, noise, and organic solvents, were obtained from baseline questionnaires and factory records at the time of enrollment (2021), reflecting current or recent exposure status (classified as yes/no).

### Statistical analyses

Continuous variables were characterized by their mean (standard deviation). Depending on whether continuous variables met assumptions of normal distribution and homogeneity of variance, various approaches were utilized for intergroup comparisons. If normality and homogeneity were met, independent samples *t*-tests or one-way analysis of variance (ANOVA) were employed for intergroup comparisons. Otherwise, the Mann–Whitney U test or Kruskal-Wallis H test was utilized. Categorical variables were characterized by frequency (%). Intergroup comparisons were conducted with the *χ*^2^ test.

Blood pressure trajectories were modeled using group-based trajectory modeling (GBTM). GBTM was performed using the gbmt package in R. Models with 1 to 5 trajectory groups were tested, assuming linear or quadratic trajectories. The optimal number of groups was selected based on the lowest Bayesian Information Criterion (BIC), an average posterior probability ≥0.7 for each group, a correct classification probability >0.7, and each group comprising at least 5% of the sample (17, 18). Trajectory groups were labeled based on their patterns. Pearson correlation analysis was used to evaluate the association among various blood pressure trajectories, with the analysis derived from the posterior probability of persons inside each trajectory.

This study initiated follow-up on January 1, 2021, with all workers enrolled in the cohort before this date and without a record of ECG abnormalities. Follow-up continued until the first occurrence of ECG abnormalities in the study subject or the end of the follow-up period (December 31, 2024), whichever occurred first. Cox proportional hazards regression analysis was used to evaluate the association between blood pressure trajectories and the risk of incident ECG abnormalities. The proportional hazards assumption was tested using Schoenfeld residuals; no violation was detected (global *p* > 0.05). Results were displayed as hazard ratios (HR) accompanied by their 95% confidence intervals (95% CI). We fitted three models: Model 1 was unadjusted; Model 2 adjusted for age group and gender; Model 3 additionally adjusted for years of service, job type, company size, ownership type, work location, smoking status, exposure to high temperatures, noise, organic solvents, and dust.

To evaluate potential effect modification, stratified analyses were performed according to occupational exposures, including high temperature, noise, organic solvents, and dust. Interaction terms between blood pressure trajectories and each exposure variable were included in the Cox regression (Model 3), and the significance of interaction was assessed using likelihood ratio tests.

All statistical analyses were conducted using R software (version 4.4.1). A two-tailed *p*-value of less than 0.05 was considered statistically significant.

### Ethics approval

The study protocol received approval from the Scientific and Medical Ethical Committee of Guangdong Province Hospital for Occupational Disease Prevention and Treatment (GDHOD MEC 2024072), and all procedures were conducted in compliance with the Declaration of Helsinki.

## Results

### Baseline characteristics

A total of 2,561 workers were included, with a mean age of 34.65 ± 7.24 years, of whom 93.44% were men. During the 4-year follow-up, at least one ECG abnormality was documented in 1,183 of the 2,561 participants. The annual detection rates ranged from 10.9 to 12.2% across the follow-up period (Supplementary Table S1). Among all ECG abnormalities, non-specific ECG changes were the most frequent category (*n* = 651, 55.0% of all ECG abnormalities), followed by irregular heartbeat (*n* = 290, 24.5%), bradycardia (*n* = 111, 9.4%), tachycardia (*n* = 67, 5.7%), conduction block (*n* = 39, 3.3%), premature contractions (*n* = 18, 1.5%), pre-excitation syndrome (*n* = 6, 0.5%), and atrial fibrillation (*n* = 1, 0.1%). The distribution of each category is detailed in the revised Supplementary Figure S1.

Compared with the normal group, the incident ECG abnormalities group had a significantly higher proportion of younger workers (aged 20–29 years), manufacturing workers, and current smokers. Regarding occupational exposures, the ECG abnormality group had significantly higher rates of dust but a lower rate of high temperature exposure compared with the normal group (all *p* < 0.05). No significant differences were observed between the two groups in terms of gender, company size, or organic solvent exposure (Supplementary Table S3).

### Identification and distribution of blood pressure trajectories

**Figure 2:**
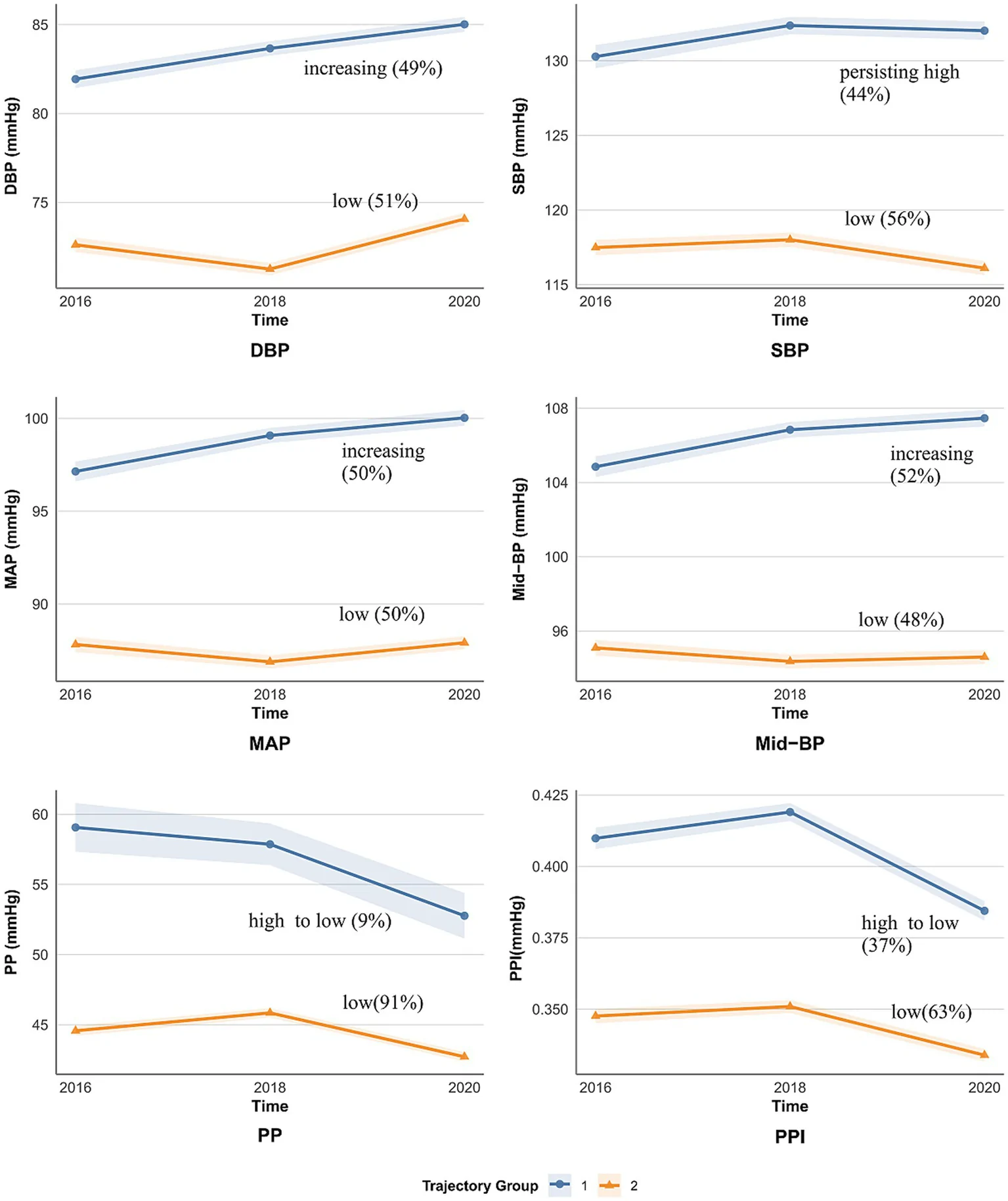
Trajectories of blood pressure.

We found that DBP, MAP, and Mid-BP shared similar patterns, characterized by low and increasing trajectories. SBP presented low and persisting high trajectories, whereas PP and PPI demonstrated low and high to low trajectories, respectively (Figure 2).

Compared to the normal group, the ECG abnormalities group exhibited significantly greater proportions of workers in the increasing trajectory groups of DBP, MAP, and Mid-BP, as well as in the SBP persisting high trajectory group (*p* < 0.001). However, the distributions of PP and PPI trajectory types exhibited no statistically significant variations between the two groups (Supplementary Table S3).

Correlation analysis (Supplementary Figure S2) revealed strong correlations among SBP, DBP, MAP, and Mid-BP trajectories (*r* > 0.70). In contrast, the trajectories of PP and PPI showed weaker associations (*r* < 0.30).

### Association between blood pressure trajectories and ECG abnormalities risk

In the unadjusted model, increasing trajectories of DBP (HR = 1.36, 95% CI: 1.21–1.52), MAP (HR = 1.34, 95% CI: 1.19–1.50), and Mid-BP (HR = 1.39, 95% CI: 1.24–1.57), as well as the persisting high SBP trajectory (HR = 1.32, 95% CI: 1.18–1.48), were all associated with an increased risk of ECG abnormalities compared with their respective low trajectories. These associations remained significant after adjustment for age and gender. In the fully adjusted Model 3, workers in the persisting high SBP trajectory (HR = 1.17, 95% CI: 1.04–1.31) had higher risk of ECG abnormalities compared to workers in the low SBP trajectory. Similarly, workers in the increasing Mid-BP trajectory (HR = 1.17, 95% CI: 1.04–1.32) had a significant risk of ECG abnormalities compared with those in the low trajectory. No significant associations were observed for PP or PPI trajectories across all three models (Table 1).

**Table 1:** Trajectories groups and the risk of ECG abnormalities.

Trajectory groups	Cases/*N* (%)	Model 1	Model 2	Model 3
DBP trajectory				
Low	540/1299 (41.6%)	1 (Ref.)	1 (Ref.)	1 (Ref.)
Increasing	643/1262 (50.9%)	1.36 (1.21–1.52)	1.34 (1.19–1.50)	1.11 (0.99–1.26)
SBP trajectory				
Low	608/1435 (42.4%)	1 (Ref.)	1 (Ref.)	1 (Ref.)
Persisting high	575/1126 (51.1%)	1.32 (1.18–1.48)	1.29 (1.15–1.45)	1.17 (1.04–1.31)
MAP trajectory				
Low	531/1273 (41.7%)	1 (Ref.)	1 (Ref.)	1 (Ref.)
Increasing	681/1288 (52.9%)	1.34 (1.19–1.50)	1.32 (1.17–1.48)	1.12 (1.00–1.27)
Mid-BP trajectory				
Low	502/1231 (40.8%)	1 (Ref.)	1 (Ref.)	1 (Ref.)
Increasing	681/1330 (51.2%)	1.39 (1.24–1.57)	1.37 (1.22–1.54)	1.17 (1.04–1.32)
PP trajectory				
Low	1062/2325 (45.7%)	1 (Ref.)	1 (Ref.)	1 (Ref.)
High to low	121/236 (51.3%)	1.18 (0.98–1.42)	1.15 (0.95–1.39)	1.20 (1.00–1.45)
PPI trajectory				
Low	758/1597 (47.5%)	1 (Ref.)	1 (Ref.)	1 (Ref.)
High to low	425/964 (44.1%)	0.90 (0.80–1.01)	0.89 (0.79–1.00)	1.03 (0.91–1.16)

### Stratified analyses

**Figure 3:**
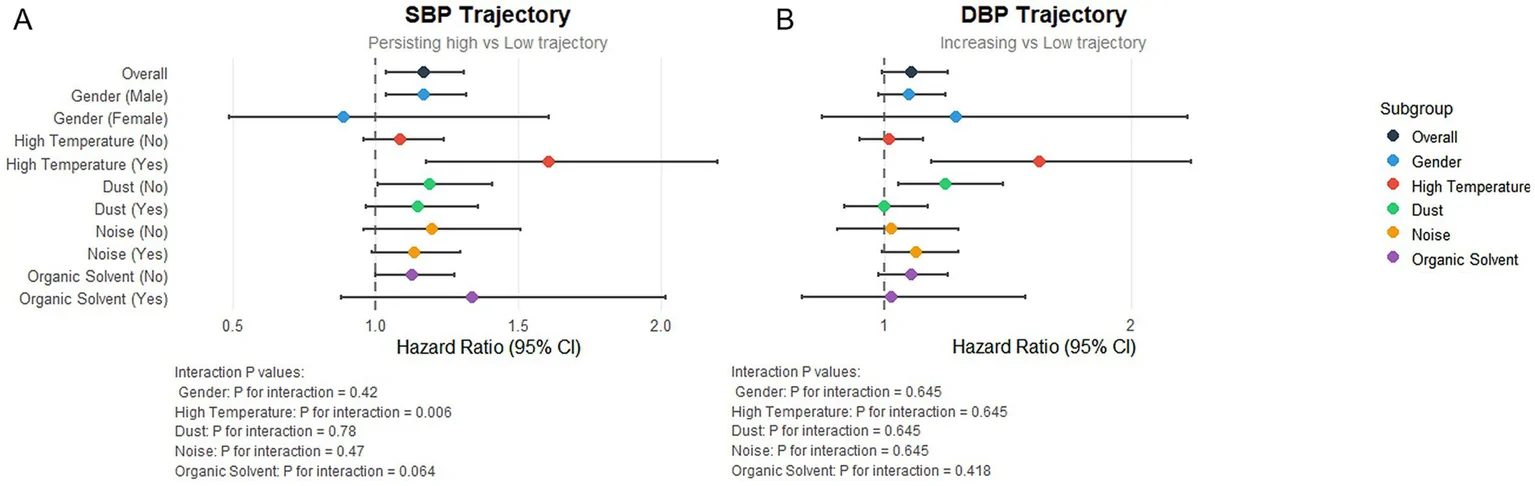
Subgroup analysis of the association between blood pressure trajectories and ECG abnormalities risk. **(A)** Diastolic blood pressure (DBP) trajectories. **(B)** Systolic blood pressure (SBP) trajectories.

Stratified analyses based on the fully adjusted model suggested that neither organic solvent nor noise exposure modified the association between any blood pressure trajectory and ECG abnormalities risk. Compared with workers unexposed to high temperatures, those exposed to high temperatures had a higher risk of ECG abnormalities associated with the persisting high SBP trajectory (HR = 1.61, 95% CI: 1.18–2.20), the increasing MAP trajectory (HR = 1.58, 95% CI: 1.15–2.16), and the increasing Mid-BP trajectory (HR = 1.42, 95% CI: 1.04–1.94) (all interaction *p* values < 0.05; Figure 3; Supplementary Table S4). Significant interactions were also observed for the PPI trajectory with high temperature and dust exposure (*p* = 0.011 for both, Supplementary Table S4).

## Discussion

This study provides evidence from a worker-based cohort on the association between long-term blood pressure trajectories and incident ECG abnormalities and further examines the modifying role of occupational exposures. Our main findings are threefold. First, six blood pressure measures each followed two distinct trajectories, with persisting high SBP and increasing Mid-BP trajectories associated with an elevated risk of ECG abnormalities. Second, high temperature exposure was associated with stronger effects of persistently high SBP and increasing Mid-BP trajectories on ECG abnormalities. Third, SBP, DBP, MAP, and Mid-BP trajectories showed strong intercorrelations.

Our finding that a persisting high SBP trajectory is associated with increased ECG abnormalities risk aligns with previous reports from the Tromsø Study, which demonstrated that a consistently increasing SBP trajectory heightens the risk of atrial fibrillation (19). The proposed mechanism involves prolonged elevated systolic pressure leading to left ventricular hypertrophy, reduced myocardial compliance, and impaired diastolic function, thereby promoting electrical instability in the heart (20, 21). Notably, our study extends previous findings by showing that Mid-BP trajectories, a composite measure of SBP and DBP, were also associated with the risk of incident ECG abnormalities, an area that has received limited attention in previous studies.

We found no significant association between DBP trajectories and ECG abnormalities risk, a finding consistent with the Framingham Heart Study, which also reported no association after controlling for multiple confounders (13). This null finding suggests that DBP trajectories may have limited predictive value for ECG abnormalities specifically in young and middle-aged working populations. Similarly, PP and PPI trajectories were not associated with ECG abnormalities risk. The Tromsø Study observed an association between elevated pulse pressure and AF risk in women but not in men (12), suggesting sex-specific effects. Given the predominantly male composition of our cohort (93.4%), we cannot rule out the possibility that the null findings for PP and PPI may be partly attributable to sex-related differences in pulsatile hemodynamics (22).

The distinct patterns of association across BP measures were further supported by correlation analysis. The strong correlations observed among SBP, DBP, MAP, and Mid-BP trajectories (*r* > 0.70) support the notion that these measures collectively reflect the sustained pressure load on the vascular wall throughout the cardiac cycle. In contrast, the weaker correlations of PP and PPI trajectories with other BP measures (*r* < 0.30) suggest that these indices capture the pulsatile component of blood pressure, which may be governed by different hemodynamic mechanisms, such as arterial stiffness and large artery buffering function (23–25). This distinction may partly explain why PP and PPI trajectories were not significantly associated with ECG abnormalities risk in our study.

A striking finding of our study is the modifying effect of occupational high temperature exposures on the association between blood pressure trajectories and ECG abnormalities risk, with interaction HRs of 1.61 for SBP and 1.42 for Mid-BP. This is biologically plausible, as extreme heat exposure triggers peripheral vasodilation, electrolyte imbalance, and accelerated heart rate, collectively increasing cardiac workload and diminishing electrophysiological stability (26). A meta-analysis reported that a 1 °C increase in ambient temperature correlates with a 1.6% rise in arrhythmia risk (27), which may contribute to an increased risk of ECG abnormalities.

This study has several strengths, including the use of high-quality longitudinal data from a multi-city occupational cohort, application of GBTM to capture dynamic blood pressure patterns, and the novel examination of occupational exposures as effect modifiers. However, several limitations warrant consideration. First, residual confounding from unmeasured factors (e.g., antihypertensive treatment, other cardiovascular medications, body mass index, diabetes, alcohol consumption) cannot be completely excluded. Younger workers (aged 20–29 years) accounted for a large proportion of ECG abnormality cases, and unmeasured lifestyle factors may have partly contributed to this pattern. Second, ECG abnormalities cases were identified through routine health examination data, precluding subclassification into specific types (e.g., AF, ventricular arrhythmias). Third, the generalizability of our findings is limited by the predominantly male (93.4%) and geographically restricted (Guangdong Province) sample. Future studies should include female workers and diverse occupational settings to validate and extend our findings. Fourth, we lacked data on reasons for retirement, resignation, or death. However, because eligibility required continuous employment through 2016–2024, no participants were censored due to these events. Nonetheless, this may have introduced a healthy worker survivor effect, which we acknowledge as a limitation. Despite these limitations, our findings have important implications for workplace cardiovascular prevention. Regular monitoring of SBP and Mid-BP trajectories among factory workers exposed to high temperatures could facilitate early identification of at-risk individuals and enable timely preventive interventions.

## Conclusion

This study finds that the persisting high SBP trajectory and increasing Mid-BP trajectory may be associated with the development of ECG abnormalities, particularly in high temperature work environments. These data suggest that regular monitoring of SBP and Mid-BP trajectories could serve as a screening tool to identify high-risk individuals for targeted workplace surveillance and lifestyle interventions. Future intervention studies are warranted to determine whether modifying these trajectories reduces ECG abnormalities risk.

### Patient and public involvement

The public was not involved in the design, conduct, or reporting of this research. There are no plans to disseminate the findings directly to participants.

## Data Availability

The datasets presented in this article are not readily available because of privacy and ethical restrictions. Requests to access the datasets should be directed to the corresponding author.
