# Supplementary material for: Trajectories of blood pressure measures and the incidence of electrocardiographic abnormalities: a worker-based cohort study
Source: Front Public Health. 2026 Sep 10;14:1930829. doi: 10.3389/fpubh.2026.1930829 (PMC13601335; doi:10.3389/fpubh.2026.1930829)
Supplement: Supplementary file 1 [file Supplementary_file_1.docx]

**Supplemental material：Trajectories of blood pressure measures and the incidence of electrocardiographic abnormalities: a worker-based cohort study**

Jiahui Liang^a,b^, Peitian Gao^d^, Xuehong Liang^a,b^, Jiachun Jin^a^, Yan Jiang^e^, Weijie Jiang^b^, Chuifei Zhong^c^, Yongshun Huang^a,*^

**Summary of contents**

**Supplementary Table and Figures**

**Table S1** Annual ECG examination completion and detection rates

**Table S2** Baseline characteristics of included and excluded participants

**Table S3** Baseline characteristics of the study population

**Table S4** Stratified analyses of blood pressure trajectories and incident ECG abnormalities (MAP, Mid-BP, PP, PPI)

**Figure S1** Distribution of ECG abnormalities (2021–2024)

**Figure S2** Correlation of blood pressure trajectories.

**Table S1** Annual ECG examination completion and detection rates

| **Year** | **Total participants** | **Completed ECG, n (%)** | **Annual detection rate* (%)** |
| --- | --- | --- | --- |
| 2021 | 2,561 | 1,744 (68.1%) | 12.20% |
| 2022 | 2,561 | 1,746 (68.2%) | 10.90% |
| 2023 | 2,561 | 1,956 (76.4%) | 11.20% |
| 2024 | 2,561 | 2,545 (99.4%) | 12.10% |

*Annual detection rate = first-ever abnormal ECG findings / at-risk population

**Table S2** Baseline characteristics of included and excluded participants

| Groups | included | excluded | P value |
| --- | --- | --- | --- |
| N | 2561 | 3252 |  |
| Age, mean (SD), years | 34.65 (7.24) | 34.55 (7.28) | 0.06 |
| Gender(%) |  |  | 0.35 |
| Male | 2393 (93.44) | 3057 (94.01) |  |
| Female | 168 ( 6.56) | 195( 5.99) |  |
| Years of service, mean (SD), years | 14.25 (5.46) | 14.43 (5.33) | 0.2 |
| Job type(%) |  |  | <0.001 |
| Management | 34 ( 1.33) | 86(2.64) |  |
| Social service worker | 166 ( 6.48) | 158 ( 4.86) |  |
| Manufacturing worker | 867 (33.85) | 834 (25.65) |  |
| Professional technical worker | 436 (17.02) | 566 (17.40) |  |
| Other | 1058 (41.31) | 1608 (48.45) |  |
| Company size(%) |  |  | 0.93 |
| Small | 434 (16.95) | 553 (17.01) |  |
| Medium | 383 (14.96) | 497 (15.28) |  |
| Large | 1744 (68.10) | 2,201 (67.69) |  |
| Ownership type(%) |  |  | < 0.001 |
| Public ownership | 652 (25.46) | 596 (18.32) |  |
| Non-public ownership | 281 (10.97) | 366 (11.25) |  |
| Mixed ownership | 1061 (41.43) | 1,376 (42.31) |  |
| Other | 567 (22.14) | 914 (28.12) |  |
| Work location |  |  | 0.008 |
| Urban | 1194 (46.62) | 1402 (43.11) |  |
| Rural | 1367 (53.38) | 1850 (56.89) |  |
| Smoking status(%) |  |  | 0.13 |
| Never | 1563 (61.03) | 1958 (60.23) |  |
| Former | 87 ( 3.40) | 144 (4.44) |  |
| Current | 911 (35.57) | 1150 (35.33) |  |
| High temperature exposure(%) |  |  | 0.73 |
| Yes | 707 (27.61) | 911 (28.01) |  |
| No | 1854 (72.39) | 2341 (71.99) |  |
| Noise exposure(%) |  |  | 0.008 |
| Yes | 1934 (75.52) | 2355 (72.43) |  |
| No | 627 (24.48) | 897 (27.57) |  |
| Organic solvent exposure(%) |  |  | 0.89 |
| Yes | 244 ( 9.53) | 314 (9.64) |  |
| No | 2317 (90.47) | 2938 (90.36) |  |
| Dust exposure(%) |  |  | < 0.001 |
| Yes | 1010 (39.44) | 1633 (50.23) |  |
| No | 1551 (60.56) | 1619 (49.77) |  |

**Table S3** Baseline characteristics of the study population

| Groups | Overall | Normal | ECG abnormalities | P value |
| --- | --- | --- | --- | --- |
| N(%) | 2561 | 1378(53.81) | 1183(46.19) |  |
| Age, mean (SD), years | 34.65 (7.24) | 34.88 (6.87) | 34.40 (7.65) | 0.09 |
| Age group, years, (%) |  |  |  | <0.001 |
| 20-29 | 666 (26.01) | 298 (21.63) | 368 (31.11) |  |
| 30-39 | 1309 (51.11) | 771 (55.95) | 538 (45.48) |  |
| 40-49 | 485 (18.94) | 262 (19.01) | 223 (18.85) |  |
| 50-59 | 101 ( 3.94) | 47 ( 3.41) | 54 ( 4.56) |  |
| Gender(%) |  |  |  | 0.32 |
| Male | 2393 (93.44) | 1281 (92.96) | 1112 (94.00) |  |
| Female | 168 ( 6.56) | 97 ( 7.04) | 71 ( 6.00) |  |
| Years of service, mean (SD), years | 14.25 (5.46) | 14.08 (5.22) | 14.44 (5.73) | 0.10 |
| Job type(%) |  |  |  | <0.001 |
| Management | 34 ( 1.33) | 20 ( 1.45) | 14 ( 1.18) |  |
| Social service worker | 166 ( 6.48) | 87 ( 6.31) | 79 ( 6.68) |  |
| Manufacturing worker | 867 (33.85) | 390 (28.30) | 477 (40.32) |  |
| Professional technical worker | 436 (17.02) | 225 (16.33) | 211 (17.84) |  |
| Other | 1058 (41.31) | 656 (47.61) | 402 (33.98) |  |
| Company size(%) |  |  |  | 0.14 |
| Small | 434 (16.95) | 250 (18.14) | 184 (15.55) |  |
| Medium | 383 (14.96) | 195 (14.15) | 188 (15.89) |  |
| Large | 1744 (68.10) | 933 (67.71) | 811 (68.55) |  |
| Ownership type(%) |  |  |  | <0.001 |
| Public ownership | 652 (25.46) | 448 (32.51) | 204 (17.24) |  |
| Non-public ownership | 281 (10.97) | 144 (10.45) | 137 (11.58) |  |
| Mixed ownership | 1061 (41.43) | 493 (35.78) | 568 (48.01) |  |
| Other | 567 (22.14) | 293 (21.26) | 274 (23.16) |  |
| Work location |  |  |  | <0.001 |
| Urban | 1194 (46.62) | 553 (40.13) | 641 (54.18) |  |
| Rural | 1367 (53.38) | 825 (59.87) | 542 (45.82) |  |
| Smoking status(%) |  |  |  | <0.001 |
| Never | 1563 (61.03) | 924 (67.05) | 639 (54.02) |  |
| Former | 87 ( 3.40) | 36 ( 2.61) | 51 ( 4.31) |  |
| Current | 911 (35.57) | 418 (30.33) | 493 (41.67) |  |
| High temperature exposure(%) |  |  |  | <0.001 |
| Yes | 707 (27.61) | 528 (38.32) | 179 (15.13) |  |
| No | 1854 (72.39) | 850 (61.68) | 1004 (84.87) |  |
| Noise exposure(%) |  |  |  | 0.001 |
| Yes | 1934 (75.52) | 1077 (78.16) | 857 (72.44) |  |
| No | 627 (24.48) | 301 (21.84) | 326 (27.56) |  |
| Organic solvent exposure(%) |  |  |  | 0.33 |
| Yes | 244 ( 9.53) | 139 (10.09) | 105 ( 8.88) |  |
| No | 2317 (90.47) | 1239 (89.91) | 1078 (91.12) |  |
| Dust exposure(%) |  |  |  | <0.001 |
| Yes | 1010 (39.44) | 435 (31.57) | 575 (48.61) |  |
| No | 1551 (60.56) | 943 (68.43) | 608 (51.39) |  |
| DBP trajectory (%) |  |  |  | <0.001 |
| Increasing | 1262 (49.28) | 619 (44.92) | 643 (54.35) |  |
| Low | 1299 (50.72) | 759 (55.08) | 540 (45.65) |  |
| SBP trajectory (%) |  |  |  | <0.001 |
| Persisting high | 1126 (43.97) | 551 (39.99) | 575 (48.61) |  |
| Low | 1435 (56.03) | 827 (60.01) | 608 (51.39) |  |
| MAP trajectory (%) |  |  |  | <0.001 |
| Increasing | 1288 (50.29) | 636 (46.15) | 652 (55.11) |  |
| Low | 1273 (49.71) | 742 (53.85) | 531 (44.89) |  |
| Mid-BP trajectory (%) |  |  |  | <0.001 |
| Increasing | 1330 (51.93) | 649 (47.10) | 681 (57.57) |  |
| Low | 1231 (48.07) | 729 (52.90) | 502 (42.43) |  |
| PP trajectory (%) |  |  |  | 0.11 |
| High to low | 236 ( 9.22) | 115 ( 8.35) | 121 (10.23) |  |
| Low | 2325 (90.78) | 1263 (91.65) | 1062 (89.77) |  |
| PPI trajectory (%) |  |  |  | 0.10 |
| High to low | 964 (37.64) | 539 (39.11) | 425 (35.93) |  |
| Low | 1597 (62.36) | 839 (60.89) | 758 (64.07) |  |

**Table S4** Stratified analyses of blood pressure trajectories and incident ECG abnormalities（MAP, Mid-BP, PP, PPI)

| **MAP trajectory: increasing VS low trajectory** | | | | | | |
| --- | --- | --- | --- | --- | --- | --- |
| Subgroup | Level | N | Events | HR (95% CI) | P value | P for interaction |
| Overall | All | 2561 | 1183 | 1.12 (1.00-1.27) | 0.059 | — |
| Gender | Male | 2393 | 1112 | 1.12 (0.99-1.27) | 0.064 | 0.763 |
|  | Female | 168 | 71 | 1.09 (0.62-1.93) | 0.766 |  |
| High‑temperature exposure | No | 1854 | 1004 | 1.04 (0.91-1.18) | 0.559 | 0.002 |
|  | Yes | 707 | 179 | 1.58 (1.15-2.16) | 0.005 |  |
| Dust exposure | No | 1551 | 608 | 1.19 (1.01-1.40) | 0.042 | 0.422 |
|  | Yes | 1010 | 575 | 1.06 (0.89-1.26) | 0.501 |  |
| Noise exposure | No | 627 | 326 | 1.19 (0.93-1.50) | 0.161 | 0.355 |
|  | Yes | 1934 | 857 | 1.09 (0.94-1.25) | 0.246 |  |
| Organic solvent exposure | No | 2317 | 1078 | 1.10 (0.97-1.25) | 0.128 | 0.144 |
|  | Yes | 244 | 105 | 1.24 (0.80-1.94) | 0.338 |  |
| **Mid-BP trajectory: increasing vs low trajectory** | | | | | | |
| Subgroup | Level | N | Events | HR (95% CI) | P value | P for interaction |
| Overall | All | 2561 | 1183 | 1.17 (1.04-1.32) | 0.01 | — |
| Gender | Male | 2393 | 1112 | 1.17 (1.04-1.33) | 0.012 | 0.709 |
|  | Female | 168 | 71 | 1.18 (0.68-2.07) | 0.554 |  |
| High‑temperature exposure | No | 1854 | 1004 | 1.11 (0.97-1.26) | 0.118 | 0.025 |
|  | Yes | 707 | 179 | 1.42 (1.04-1.94) | 0.028 |  |
| Dust exposure | No | 1551 | 608 | 1.22 (1.04-1.44) | 0.018 | 0.565 |
|  | Yes | 1010 | 575 | 1.13 (0.95-1.35) | 0.165 |  |
| Noise exposure | No | 627 | 326 | 1.15 (0.91-1.46) | 0.246 | 0.814 |
|  | Yes | 1934 | 857 | 1.16 (1.01-1.34) | 0.037 |  |
| Organic solvent exposure | No | 2317 | 1078 | 1.16 (1.02-1.32) | 0.021 | 0.32 |
|  | Yes | 244 | 105 | 1.16 (0.75-1.80) | 0.5 |  |
| **PP trajectory: high to low vs low trajectory** | | | | | | |
| Subgroup | Level | N | Events | HR (95% CI) | P value | P for interaction |
| Overall | All | 2561 | 1183 | 1.20 (1.00-1.45) | 0.056 | — |
| Gender | Male | 2393 | 1112 | 1.22 (1.01-1.48) | 0.044 | 0.399 |
|  | Female | 168 | 71 | 0.49 (0.14-1.74) | 0.271 |  |
| High‑temperature exposure | No | 1854 | 1004 | 1.23 (1.00-1.51) | 0.048 | 0.538 |
|  | Yes | 707 | 179 | 1.01 (0.61-1.67) | 0.972 |  |
| Dust exposure | No | 1551 | 608 | 1.13 (0.88-1.46) | 0.346 | 0.419 |
|  | Yes | 1010 | 575 | 1.28 (0.96-1.70) | 0.093 |  |
| Noise exposure | No | 627 | 326 | 1.33 (0.90-1.97) | 0.147 | 0.501 |
|  | Yes | 1934 | 857 | 1.16 (0.93-1.44) | 0.182 |  |
| Organic solvent exposure | No | 2317 | 1078 | 1.19 (0.98-1.46) | 0.08 | 0.955 |
|  | Yes | 244 | 105 | 1.17 (0.59-2.33) | 0.648 |  |
| **PPI trajectory: high to low vs low trajectory** | | | | | | |
| Subgroup | Level | N | Events | HR (95% CI) | P value | P for interaction |
| Overall | All | 2561 | 1183 | 1.03 (0.91-1.16) | 0.665 | — |
| Gender | Male | 2393 | 1112 | 1.05 (0.92-1.19) | 0.473 | 0.144 |
|  | Female | 168 | 71 | 0.60 (0.32-1.10) | 0.099 |  |
| High‑temperature exposure | No | 1854 | 1004 | 1.10 (0.96-1.25) | 0.174 | 0.011 |
|  | Yes | 707 | 179 | 0.79 (0.58-1.07) | 0.128 |  |
| Dust exposure | No | 1551 | 608 | 0.88 (0.75-1.05) | 0.148 | 0.011 |
|  | Yes | 1010 | 575 | 1.19 (1.00-1.42) | 0.051 |  |
| Noise exposure | No | 627 | 326 | 1.05 (0.82-1.34) | 0.7 | 0.608 |
|  | Yes | 1934 | 857 | 1.02 (0.88-1.17) | 0.821 |  |
| Organic solvent exposure | No | 2317 | 1078 | 1.04 (0.92-1.18) | 0.516 | 0.476 |
|  | Yes | 244 | 105 | 0.92 (0.60-1.43) | 0.712 |  |

**Figure S1** Distribution of ECG abnormalities (2021–2024)

**Figure S2** Correlation of blood pressure trajectories.
